# Low-density lipoprotein cholesterol to high-density lipoprotein cholesterol ratio is the best surrogate marker for insulin resistance in non-obese Japanese adults

**DOI:** 10.1186/1476-511X-9-138

**Published:** 2010-12-07

**Authors:** Ryuichi Kawamoto, Yasuharu Tabara, Katsuhiko Kohara, Tetsuro Miki, Tomo Kusunoki, Shuzo Takayama, Masanori Abe, Tateaki Katoh, Nobuyuki Ohtsuka

**Affiliations:** 1 Department of Community Medicine, Ehime University Graduate School of Medicine; Ehime 791-0295, Japan; 2 Geriatric Medicine, Ehime University Graduate School of Medicine; Ehime 791-0295, Japan; 3 Department of Internal Medicine, Seiyo Municipal Nomura Hospital, Ehime 797-1212, Japan

## Abstract

**Background:**

The aim of the present study was to examine how lipid profiles are associated with insulin resistance in Japanese community-dwelling adults.

**Methods:**

This cross-sectional study included 614 men aged 58 ± 14 (mean ± standard deviation; range, 20-89) years and 779 women aged 60 ± 12 (range, 21-88) years. The study sample were 1,042 (74.8%) non-obese (BMI < 25.0 kg/m^2^) and 351 (25.2%) overweight (BMI ≥ 25 kg/m^2^) subjects. Insulin resistance was defined by homeostasis model assessment of insulin resistance (HOMA-IR) of at least 2.5. The areas under the curve (AUC) of the receiver operating characteristic curves (ROC) were used to compare the power of these serum markers.

**Results:**

In non-obese subjects, the best marker of insulin resistance was low-density lipoprotein cholesterol (LDL-C)/high-density lipoprotein cholesterol (HDL-C) ratio of 0.74 (95% confidence interval (CI), 0.66-0.80). The HDL-C, triglyceride (TG)/HDL-C ratio, and non-HDL-C also discriminated insulin resistance, as the values for AUC were 0.31 (95% CI, 0.24-0.38), 0.69 (95% CI, 0.62-0.75) and 0.69 (95% CI, 0.62-0.75), respectively. In overweight subjects, the AUC for TG and TG/HDL-C ratio were 0.64 (0.58-0.71) and 0.64 (0.57-0.70), respectively. The optimal cut-off point to identifying insulin resistance for these markers yielded the following values: TG/HDL-C ratio of ≥1.50 and LDL-C/HDL-C ratio of ≥2.14 in non-obese subjects, and ≥2.20, ≥2.25 in overweight subjects. In non-obese subjects, the positive likelihood ratio was greatest for LDL-C/HDL-C ratio.

**Conclusion:**

In non-obese Japanese adults, LDL-C/HDL-C ratio may be the best reliable marker of insulin resistance.

## Background

Being overweight is also a major worldwide public health problem. In Japanese adults, it was demonstrated that 29.3% of men and 19.5% of women are overweight (body mass index (BMI); weight (kg)/height (m) ^2^, ≥25 kg/m^2^), with approximately 3.3% of men and 3.2% of women designated as obese (BMI ≥ 30 kg/m^2^) [1]. Several large-scale studies have shown that overweight people, defined on the basis of a high BMI, increases the risk of hypertension, dyslipidemia, diabetes and developing cardiovascular disease (CVD) such as stroke and myocardial infarction than subjects with normal BMI [2-4]. Moreover, it has been suggested that individuals exist who are not overweight, but who, like people with overt obesity, also have hyperinsulinemia, are insulin resistant, and are predisposed to hypertriglyceridemia, type 2 diabetes mellitus, and premature cardiovascular disease [5,6]. Much investigation has been focused on insulin resistance as the primary determinant of atherosclerotic risk factors, independent of BMI [7].

On the other hand, insulin resistant persons also have a characteristic dyslipidemia [8] and measuring these variables might help identify insulin resistance. Plasma triglycerides, high-density lipoprotein cholesterol (HDL-C) [9,10], and total cholesterol (T-C)/HDL-C are independently associated with insulin resistance and risk factors of CVD [11]. However, in Japanese community-dwelling persons, there are few studies to demonstrate a relationship between lipid profiles and insulin resistance, categorized by BMI.

We took advantage of the large representative sample of Japanese adults who participated at the time of their annual health examination. We investigated how lipid profiles were associated with insulin resistance in healthy Japanese adults. For this, we used cross-sectional data from community-dwelling participants without clinical diabetes.

## Methods

### Subjects

Participants were recruited at the time of their annual health examination in a rural town with a total population of 11,136 (as of April 2002) and located in Ehime prefecture, Japan, in 2002. Among the 9,133 adults (4,395 of them male) aged 19 to 90 years in this population, a random sample of 3,164 subjects (34.6%) was recruited. Information on medical history, present conditions, and drugs was obtained by interview. Other characteristics, e.g., smoking and alcohol habits, and medication, were investigated by individual interviews using a structured questionnaire. Subjects taking medications for hypertension, diabetes, or dyslipidemia were excluded. The final study sample included 614 men and 779 women. This study was approved by the ethics committee of Ehime University School of Medicine, and written informed consent was obtained from each subject.

### Evaluation of confounding factors

Information on demographic characteristics and risk factors was collected using clinical files. Body mass index was calculated by dividing weight (in kilograms) by the square of the height (in meters). We measured blood pressure in the right upper arm of participants in a sedentary position using an automatic oscillometric blood pressure recorder (BP-103i; Colin, Aichi, Japan) while the subjects were seated after having rested for at least 5 min. Smoking status was defined as the number of cigarette packs per day multiplied by the number of years smoked (pack·year), and the participants were classified into never smokers, past smokers, light smokers (<30 pack·year) and heavy smokers (≥30 pack·year). The daily alcohol consumption was measured using the Japanese liquor unit in which a unit corresponds to 22.9 g of ethanol, and the participants were classified into never drinkers, occasional drinkers (<1 unit/day), light drinkers (1-1.9 unit/day), and heavy drinkers (≥2 unit/day). T-C, TG, HDL-C, fasting blood glucose (FBG), creatinine (enzymatic method), uric acid, and immunoreactive insulin (IRI), and high molecular weight (HMW) adiponectin (FUJIREBIO, Tokyo, Japan) were measured during fasting low-density lipoprotein cholesterol (LDL-C) levels were calculated using the Friedewald formula [12]. Participants with TG levels ≥400 mg/dl were excluded. Homeostasis of minimal assessment of insulin resistance (HOMA-IR) was calculated from FBG and IRI levels using the following formula; {FBG (mg/dL) × IRI (mU/mL)}/405 [13], and a borderline level of Insulin resistance was defined as HOMA-IR ≥ 1.6 [13], and a definite level, HOMA-IR ≥ 2.5 [14].

### Statistical analysis

Statistical analysis was performed using PASW Statistics 17.0 (Statistical Package for Social Science Japan, Inc., Tokyo, Japan). All values are expressed as mean ± standard deviation (SD), unless otherwise specified. Data for TG, FBG, HOMA-IR, and serum HMW adiponectin were skewed, and log-transformed for analysis. Subjects were divided into two groups based on BMI (non-obese, <25.0 kg/m^2^; overweight, ≥25.0 kg/m^2^), and differences between the two groups were determined by Student's t-test andχ^2 ^test. In addition, areas under the receiver operating characteristic (ROC) curves were determined for each variable to identify the predictors of insulin resistance. Areas under the ROC curves are provided with standard errors. An ROC curve is a plot of the sensitivity (true positive) versus 1-specificity (false positive) for each potential marker tested. The area under the ROC curve is a summary of the overall diagnostic accuracy of the test. The best markers have ROC curves that are shifted to the left with areas under the curve near unity. Nondiagnostic markers are represented by diagonals with areas under the ROC curves close to 0.5. Likelihood ratios were calculated as the ratios of sensitivity - (1 - specificity) (positive likelihood ratio) and (1 - sensitivity) - specificity (negative likelihood ratio). A value of *P *< 0.05 was considered significant.

## Results

### Background factors of subjects categorized by BMI

Table 1 shows the value of each background factor categorized by BMI. The subjects comprised 614 men aged 58 ± 14 (mean ± standard deviation; range, 20-89) years and 779 women aged 60 ± 12 (range, 21-88) years. The mean BMI in the study sample was 23.1 ± 3.1 kg/m^2^, with 1,042 non-obese (BMI < 25.0 kg/m^2^) (74.8%) and 351 overweight (BMI ≥ 25 kg/m^2^) (25.2%). Alcohol consumption, systolic blood pressure (SBP), diastolic blood pressure (DBP), T-C, TG, LDL-C, TG/HDL-C ratio, non-HDL-C, LDL-C/HDL-C ratio, and uric acid were significantly higher in subjects with a BMI ≥ 25.0 kg/m^2^, but age, HDL-C and serum HMW adiponectin were significantly lower in that group. There were no inter-group differences in sex, smoking status, eGFR, and prevalence of CVD.

**Table 1:** Characteristics of subjects categorized by body mass index

	Total	Non-obese	Overweight	
Body mass index† Characteristics	All N = 1,393	**<25.0 kg/m**^**2**^ N = 1,042	**≥25.0 kg/m**^**2**^ N = 351	*P *-value*
Male sex, %	44.1	43.3	46.4	0.320
Age (years)	59 ± 13	60 ± 13	57 ± 12	0.006
Body mass index (kg/m^2^)	23.1 ± 3.1	21.7 ± 2.0	27.1 ± 2.2	<0.001
Smoking status {never/ex/light/heavy (%)}	71.0/9.6/9.0/10.4	72.3/8.4/8.8/10.5	67.2/13.1/9.4/10.3	0.073
Alcohol consumption {never/light/moderate/heavy(%)}	39.0/31.7/18.6/10.8	40.7/30.3/19.0/10.0	33.9/35.6/17.4/13.1	0.043
Systolic blood pressure (mmHg)	133 ± 21	132 ± 21	139 ± 19	<0.001
Diastolic blood pressure (mmHg)	80 ± 11	79 ± 11	83 ± 11	<0.001
Total cholesterol (mg/dL)	201 ± 35	200 ± 34	204 ± 35	0.031
Triglycerides (mg/dL)	90 (68-127)	85 (64-117)	108 (81-153)	<0.001
HDL cholesterol (mg/dL)	62 ± 15	64 ± 16	57 ± 13	<0.001
LDL cholesterol (mg/dL)	118 ± 31	116 ± 31	123 ± 32	<0.001
Triglycerides/HDL cholesterol ratio	1.5 (1.0-2.3)	1.3 (0.9-2.0)	1.9 (1.3-3.2)	0.001
Non-HDL cholesterol (mg/dL)	139 ± 34	135 ± 34	148 ± 34	<0.001
LDL-cholesterol/HDL-cholesterol ratio	2.01 ± 0.78	1.92 ± 0.74	2.30 ± 0.82	<0.001
Uric acid (mg/dL)	5.0 ± 1.4	4.8 ± 1.4	5.5 ± 1.4	<0.001
eGFR	83.3 ± 16.4	83.4 ± 16.1	82.9 ± 17.2	0.659
Serum HMW adiponectin (μg/mL)	3.0 (4.9-8.0)	5.4 (3.2-8.7)	3.9 (2.2-5.8)	<0.001
Cardiovascular disease, %	4.7	4.8	4.3	0.771

HDL, high-density lipoprotein; LDL, low-density lipoprotein; eGFR, estimated glomerular filtration rate; HMW, high molecular weight. Data presented are mean ± standard deviation. Data for triglycerides, TG/HDL-C ratio, and serum adiponectin were skewed, and are presented as median (interquartile range). Data for triglycerides, TG/HDL-C ratio, and serum adiponectin were log-transformed for analysis. * Student's t-test or χ^2 ^test.

### Insulin resistance of subjects categorized by BMI

Fasting blood glucose, IRI, and HOMA-IR were significantly higher in overweight subjects (Table 2), and prevalence of insulin resistance (HOMA-IR > 1.6 or >2.5) was significantly higher in overweight subjects than in non-obese subjects.

**Table 2:** Insulin resistance of subjects categorized by body mass index

	Total	Non-obese	Overweight	
Body mass index Characteristics	All N = 1,393	**<25.0 kg/m**^**2**^ N = 1,042	**≥25.0 kg/m**^**2**^ N = 351	*P *-value*
Fasting blood glucose (mg/dL)	92 (87-99)	91 (86-98)	95 (89-103)	<0.001
Immunoreactive insulin (mU/mL)	5.1 (3.4-7.6)	4.5 (3.0-6.5)	8.0 (5.3-11.2)	<0.001
HOMA-IR§	1.19 (0.75-1.83)	1.02 (0.65-1.52)	1.91 (1.23-2.81)	<0.001
HOMA-IR§ ≤ 1.6, %	68.1	77.9	39.0	
HOMA-IR§ > 1.6 and ≤2.5, %	19.8	16.1	30.8	<0.001
HOMA-IR§ > 2.5, %	12.1	6.0	30.2	<0.001

HOMA-IR, homeostasis of minimal assessment of insulin resistance. Data for fasting blood glucose, fasting insulin, and HOMA-IR were skewed, and are presented as median (interquartile range). §HOMA-IR was calculated using the following formula; {fasting blood glucose (FBG) (mg/dL) X fasting Immunoreactive insulin (mU/mL)}/405. Data for fasting blood glucose, fasting insulin, and HOMA-IR were log-transformed for analysis. *Student's t test or χ^2 ^test.

### Comparison of areas under ROC curves (95% CI) for potential markers of insulin resistance of subjects categorized by BMI

**Figure 1:**
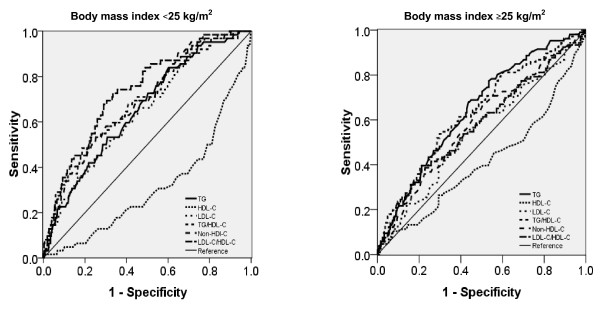
**Receiver operating characteristics (ROC) curves**. Sensitivity represents the true-positive results and 1-specificity, the false-positive results. The best markers have ROC curves that are shifted to the left with areas under the curve near unity. Nondiagnostic markers are represented by diagonals with areas under the ROC curves close to 0.5.

In non-obese subjects, the ROC curve analyses showed that the best marker of insulin resistance was LDL-C/HDL-C ratio, with an area under the ROC curve of 0.74 (0.66-0.80) (Table 3; Figure 1). The TG/HDL-C ratio, HDL-C, and non-HDL-C also discriminated insulin resistance, as they had areas under the ROC curve of 0.31 (0.24-0.38), 0.69 (0.62-0.75) and 0.69 (0.62-0.75), respectively. In overweight subjects, TG and TG/HDL-C ratio were more effective.

**Table 3:** Comparison of areas under the ROC curves (95% CI) for potential markers of insulin resistance (HOMA-IR§ > 2.5) of subjects categorized by body mass index

	AUC (95% CI)					
		
	Total	Non-obese		Overweight		
Body mass index Characteristics	All N = 1,393	*P*-value	**<25.0 kg/m**^**2**^ N = 1,042	*P*-value	**>25.0 kg/m**^**2**^ N = 351	*P*-value
Triglycerides (mg/dL)	0.69 (0.45-0.73)	<0.001	0.66 (0.59-0.72)	<0.001	0.64 (0.58-0.71)	<0.001
HDL cholesterol (mg/dL)	0.33 (0.28-0.37)	<0.001	0.31 (0.24-0.38)	<0.001	0.41 (0.35-0.48)	0.009
LDL cholesterol (mg/dL)	0.61 (0.56-0.65)	<0.001	0.65 (0.58-0.72)	<0.001	0.54 (0.48-0.61)	0.188
Triglycerides/HDL cholesterol ratio	0.70 (0.66-0.75)	<0.001	0.69 (0.62-0.75)	<0.001	0.64 (0.57-0.70)	<0.001
Non-HDL cholesterol (mg/dL)	0.66 (0.61-0.70)	<0.001	0.69 (0.62-0.75)	<0.001	0.59 (0.52-0.65)	0.011
LDL-cholesterol/HDL-cholesterol ratio	0.69 (0.65-0.73)	<0.001	0.74 (0.66-0.80)	<0.001	0.58 (0.51-0.65)	0.021

ROC, receiver operating characteristics; HOMA-IR, homeostasis of minimal assessment of insulin resistance; CI, confidence interval; AUC, area under ROC curve; HDL, high-density lipoprotein; LDL, low-density lipoprotein. §HOMA-IR was calculated using the following formula; {fasting blood glucose (mg/dL) X immuno-reactive insulin (mU/mL)}/405.

### Optimal cut-off point of TG/HDL-C ratio and LDL-C/HDL-C ratio for predicting insulin resistance of subjects categorized by BMI

Table 4 shows the cut-off points of TG/HDL-C ratio and LDL-C/HDL-C ratio for identifying insulin resistance. The optimal cut-off point to identifying insulin resistance for these markers yielded the following values: TG/HDL-C ratio of ≥1.50 and LDL-C/HDL-C ratio of ≥2.14 in non-obese subjects, and ≥2.20, ≥2.25 in overweight. In non-obese subjects, the positive likelihood ratio value indicates that the odds of insulin resistance increased by 2.30-fold if the LDL-C/HDL-C ratio was positive (the value ≥2.14). This ratio was greater for LDL-C/HDL-C ratio than TG/HDL-C ratio. The negative likelihood ratios indicate the extent to which the odds of insulin resistance decrease if the test is negative. These odds also decreased more so for LDL-C/HDL-C ratio. In overweight subjects, these values were similar.

**Table 4:** Comparison of triglyceride/HDL cholesterol ratio and LDL cholesterol/ HDL cholesterol ratio for predicting of insulin resistance (HOMA-IR§ > 2.5) of subjects categorized by body mass index

	HOMA-IR§						
Cut-off point Characteristics	≤2.5	>2.5	Sensitivity	Specificity	Positive LR	Negative LR	Accuracy
Body mass index† < 25.0 kg/m^2^	N = 980	N = 62					%
Triglycerides/HDL cholesterol ratio <1.50	580	20	0.67	0.59	1.63	0.56	59.7
Triglycerides/HDL cholesterol ratio ≥1.50	400	42					
LDL cholesterol/HDL cholesterol ratio <2.14	682	19	0.69	0.70	2.30	0.44	69.6
LDL cholesterol/HDL cholesterol ratio ≥2.14	298	43					
Body mass index† ≥ 25.0 kg/m^2^	N = 245	N = 106					
Triglycerides/HDL cholesterol ratio <2.20	157	45	0.57	0.64	1.58	0.67	62.1
Triglycerides/HDL cholesterol ratio ≥2.20	88	61					
LDL cholesterol/HDL cholesterol ratio <2.25	143	49	0.59	0.58	1.40	0.71	57.0
LDL cholesterol/HDL cholesterol ratio ≥2.25	102	57					

HOMA-IR, homeostasis of minimal assessment of insulin resistance; LR, likelihood ratio; HDL, high-density lipoprotein; LDL, low-density lipoprotein. §HOMA-IR was calculated using the following formula; (fasting blood glucose (mg/dL) X immunoreactive insulin (mU/mL))/405.

## Discussion

In the present study, we examined whether lipid profiles (i.e., TG, HDL-C, LDL-C, TG/HDL-C ratio, LDL-C/HDL-C ratio and non-HDL-C) were associated with insulin resistance in Japanese adults, categorized by body mass index. Most fundamental is the fact that not all overweight or obese persons are insulin resistant. In non-obese subjects, 6.0% of them were insulin resistant, and the best marker of insulin resistance was LDL-C/HDL-C ratio, but HDL-C, TG/HDL-C ratio and non-HDL-C also discriminated insulin resistance. In overweight subjects, the areas of TG and TG/HDL-C ratio were greater than those of the other parameters. The optimal cut-off point to identifying insulin resistance for these markers yielded the following values: TG/HDL-C ratio of ≥1.50 and LDL-C/HDL-C ratio of ≥2.14 in non-obese subjects, and ≥2.20 and ≥2.25, respectively in overweight. The positive likelihood ratio was greatest for LDL-C/HDL-C ratio in non-obese subjects. Lipid ratio of LDL-C/HDL-C might be used as an integrated and simple lipid measure to evaluate insulin resistance in non-obese subjects.

Resistance to insulin-mediated glucose disposal is distributed continuously through the general population [15], and we have no criterion with which to identify a participant as being insulin resistance or insulin sensitive. However, we classified a participant as insulin resistant if he or she was in HOMA-IR > 2.5 [14]. Previous studies have shown that HOMAIR-based insulin resistance scores strongly correlate with glucose clamp-assessed insulin resistance [13,15]. However, the validation was carried out in only a few subjects, and HOMA-IR is less accurate and precise than the glucose clamp in measuring insulin resistance, but this limitation is mitigated when the number of subjects examined is large, as in our study [16].

Hypertriglyceridemia and low HDL-C almost never occurred as isolated disorders, and were nearly always associated with insulin resistance because insulin affects very low-density lipoprotein and HDL-C metabolism [17]. In previous studies, several lipid ratios have been proposed as simple and useful clinical indicators of insulin resistance. The TG/HDL-C, the T-C/HDL-C, and the LDL-C/HDL-C ratio have shown similar potential for insulin resistance, though the reports are not entirely consistent. In 50 white Americans, both TG and TG/HDL-C ratio were acceptable markers for insulin resistance, with area under the ROC curve of 0.763 and 0.770, respectively, but poor predictors in 99 African Americans, with the values at 0.625 and 0.639, respectively [18]. It was demonstrated that the relationship between TG and TG/HDL-C with insulin resistance differs by ethnicity. In 3,014 patients (mean age 54 years; 55% women), TG/HDL-C ratio and T-C/HDL-C ratio were related to insulin resistance assessed by the top quartile of the HOMA-IR, and the area under the ROC curves for predicting insulin resistance with TG/HDL-C ratio and T-C/HDL-C ratio were 0.745 and 0.707, respectively [19]. LDL-C/HDL-C ratio [20] as well as TG/HDL-C ratio [21] have advantages for standardization and identification of patients with an atherogenic lipoprotein profile as a surrogate maker of insulin resistance. Also in our study, both LDL-C/HDL-C and TG/HDL-C ratio were useful makers of insulin resistance, especially in all subjects or non-obese subjects. However, these makers were weaker in overweight subjects. Kimm et al. [21] demonstrated that the lipid ratios of TC/HDL-C, LDL-C/HDL-C and TG/HDL-C, as well as TG and HDL-C, were consistently associated with metabolic syndrome and insulin resistance in participants without metabolic syndrome, though these relations were weaker in participants with metabolic syndrome. Lipid ratios that include information on at least two measures might have a more integrated explanation than single lipid measures such as TG or HDL-C [21].

Some limitations of this study must be considered. First, the response rate was as low as 35% that is usually the casein other conventional community studies in Japan. However, the relatively large sample size enabled the assessment of an extensive array of insulin resistances in relation to lipid profiles. Second, the cross-sectional study design is limited in its ability to eliminate causal relationships between lipid profiles and HOMA-IR. Third, our definition of HOMA-IR is based on a single assessment of FBS and IRI, which may introduce misclassification bias. Therefore the demographics and referral source may limit generalizability.

In conclusion, the present study demonstrated that special lipid profiles are associated with insulin resistance according to BMI in a general population. The ability to identify who is non-obese or overweight and who are insulin resistant could help health care professionals in bringing about lifestyle interventions. In that context, use of the cutoff-points of LDL-C/HDL-C ratio or TG/HDL-C ratio described in this report is simple and useful. The present data documented that insulin resistance was present even in subjects with BMI within the normal range. Further prospective population-based studies are needed to investigate the changes in lipid metabolism by lifestyle interventions.

## Competing interests

The authors declare that they have no competing interests.

## Authors' contributions

RK, YT, and KK participated in the design of the study, performed the statistical analysis and drafted the manuscript. NO, TaK, and ToK contributed to acquisition of data and its interpretation. ST and MA contributed to conception and design of the statistical analysis. TM conceived of the study, participated in its design, coordination and helped to draft the manuscript. All authors read and approved the manuscript.
